# Impact of Non-pharmaceutical Interventions on the Control of COVID-19 in Iran: A Mathematical Modeling Study

**DOI:** 10.34172/ijhpm.2021.48

**Published:** 2021-06-09

**Authors:** Mehran Nakhaeizadeh, Sana Eybpoosh, Yunes Jahani, Milad Ahmadi Gohari, Ali Akbar Haghdoost, Lisa White, Hamid Sharifi

**Affiliations:** ^1^Modeling in Health Research Center, Institute for Futures Studies in Health, Kerman University of Medical Sciences, Kerman, Iran.; ^2^Department of Biostatistics and Epidemiology, School of Public Health, Kerman University of Medical Sciences, Kerman, Iran.; ^3^Department of Epidemiology and Biostatistics, Research Centre for Emerging and Reemerging Infectious Diseases, Pasteur Institute of Iran, Tehran, Iran.; ^4^HIV/STI Surveillance Research Center, and WHO Collaborating Center for HIV Surveillance, Institute for Futures Studies in Health, Kerman University of Medical Sciences, Kerman, Iran.; ^5^Big Data Institute, Li Ka Shing Centre for Health Information and Discovery, Nuffield Department of Medicine, University of Oxford, Oxford, UK.

**Keywords:** COVID-19, Non-pharmaceutical Interventions, Modeling, Iran

## Abstract

**Background:** During the first months of the coronavirus disease 2019 (COVID-19) pandemic, Iran reported high numbers of infections and deaths. In the following months, the burden of this infection decreased significantly, possibly due to the impact of a package of interventions. We modeled the dynamics of COVID-19 infection in Iran to quantify the impacts of these interventions.

**Methods:** We used a modified susceptible–exposed–infected–recovered (SEIR) model to model the COVID-19 epidemic in Iran, from January 21, 2020 to September 21, 2020. We estimated the 95% uncertainty intervals (UIs) using Markov chain Monte Carlo simulation. Under different scenarios, we assessed the effectiveness of non-pharmaceutical interventions (NPIs) including physical distancing measures and self-isolation. We also estimated the time-varying reproduction number (R_t_ ), using our mathematical model and epidemiologic data.

**Results:** If no NPIs were applied, there could have been a cumulative number of 51 800 000 (95% UI: 1 910 000– 77 600 000) COVID-19 infections and 266 000 (95% UI: 119 000–476 000) deaths by September 21, 2020. If physical distancing interventions, such as school/border closures and self-isolation interventions had been introduced a week earlier than they were actually launched, 30.8% and 35.2% reduction in the number of deaths and infections respectively could have been achieved by September 21, 2020. The observed daily number of deaths showed that the Rt was one or more than one almost every day during the analysis period.

**Conclusion:** Our models suggest that the NPIs implemented in Iran between January 21, 2020 and September 21, 2020 had significant effects on the spread of the COVID-19 epidemic. Our study also showed that the timely implementation of NPIs showed a profound effect on further reductions in the numbers of infections and deaths. This highlights the importance of forecasting and early detection of future waves of infection and of the need for effective preparedness and response capabilities.

## Background

**Key Messages**

**Implications for policy makers**

- With no interventions, over 51 000 000 coronavirus disease 2019 (COVID-19) infections and, up to 4 500 000 hospitalizations, over 260 000 deaths could have occurred in Iran by September 21, 2020.
- If non-pharmaceutical interventions (NPIs) started a week earlier than the time it launched, approximately 30% reduction in the number of infections and deaths could have happened.
- Except during the period from 5 April to 10 May control reproduction number was one or higher than one.

**Implications for the public**

This is the first study conducted in Iran to evaluate the impact of non-pharmaceutical interventions (NPIs). Our study suggests that the NPIs implemented in Iran between January 21, 2020 and September 21, 2020 had a satisfactory effect on reducing the number of coronavirus disease 2019 (COVID-19) infections and deaths in the country. Therefore, it is recommended that these interventions continue to be implemented by the government. Also, it is highly recommended that innovative modifications and solutions are considered that could minimize the economic impact of these NPIs on individuals and the state. Examples may include use of mobile money and microfinance to drive small business development, strengthening insurance and other adaptation measures to enhance resilience, strengthening digitalization of businesses, and direct involvement of the government in supporting innovative solutions. Our study also highlighted the profound effect the timely implementation of NPIs can have on further reductions in the number of infections and deaths.

The coronavirus disease 2019 (COVID-19) pandemic began in China in December 2019,^1^ and the disease is now a major threat to global health, with 216 countries having reported at least one case.^2^ As of September 21, 2020, there have been 31 606 824 confirmed cases reported and 977 977 deaths worldwide. The first cases of COVID-19 in Iran were reported in Qom city (central Iran) on February 19, 2020.^3^ Currently, the disease is being reported in all provinces throughout the country.^4^ During the first months of the COVID-19 pandemic, Iran reported high numbers of infections and deaths due to COVID-19. The numbers of reported cases and deaths in Iran are the highest among the countries in the Middle East region. However, in the following months, the burden of this infection decreased significantly, possibly due to the impact of a package of interventions. September 18, 2020, the numbers of reported cases and deaths in Iran were 419 043 and 24 118, respectively.^5^ Based on COVID-19 infection rates (the number of reported cases per million population), Iran ranks seventh in the region, after Qatar, Bahrain, Kuwait, United Arab Emirates, Turkey, and Occupied Palestinian Territory.^6^

 Non-pharmaceutical interventions (NPIs) have been implemented in Iran since the early stage of the epidemic. These NPIs have included major physical distancing interventions, including school and university closures; closure of holy shrines in Mashhad, Qom, and other cities; cancelation of mass gatherings, such as sporting events and congregational prayers; travel bans; and strict economic and social lockdown. On April 19, 2020, Iran gradually implemented the termination of most of these physical distancing measures due to the economic problems they were causing. In mid-March 2020, health authorities began promoting the self-isolation of confirmed cases at home. Also, post-discharge isolation units were developed in almost all cities in Iran.^7,8^

 Mathematical models can be used to assess the impact of intervention strategies and to understand the epidemic mechanisms of infectious diseases. Several studies have investigated the implementation NPIs in order to examine the potential effect of control measures on the dynamics of COVID-19 in the different regions by using mathematical model. Stochastic susceptible–exposed–infected–recovered (SEIR) model was used to examine the impact of different NPIs on the burden of COVID-19 in the United Kingdom.^9^ Yang et al have conducted a study in New York city using an age-specific SEIR model to assess the effectiveness of different NPIs in New York City. They showed the control policies implemented reduced the number of infections and the number of death cases by 72% and 76%, respectively.^10^ Lai et al demonstrated that the efficacy of various NPIs and their timings. Using a travel network-based SEIR model, they estimated that the number of COVID-19 cases would have reduced by 66%, if NPIs would have been conducted one week earlier in China.^11^ Furthermore, A number of substantial studies have evaluated the effectiveness of NPIs to calculate the time-varying effective reproduction number.^12-17^ Time-varying effective reproduction number had downward trend after implementing of NPIs.

 To the best of our knowledge, no study has examined the impact of NPIs on the control of COVID-19 in Iran. This study aimed to evaluate the impact of NPIs introduced in Iran, using a mathematical model. We also aimed to estimate the time-varying reproduction number and assess the impact of the NPIs on this number.

## Methods

###  Mathematical Model 

 We implemented a generalized SEIR compartmental model. The model framework was introduced in previous work.^18^ In brief, our model divides individuals into susceptible (S), latent (E), infected (I), isolated (IS), dead (D), hospitalized (H), temporarily isolated in isolation units (T), and recovered (R) states (Figure S1 in Supplementary file 1). Susceptible individuals acquire infection with a force of infection $\lambda =\beta \left(t\right)C\left(t\right)\frac{II}{N}$ , where β(t) is the transmission probability, C(t) is the contact rate (per participant per day), and II is the total number of infected people who could transmit the infection (infected + (0.1 × temporarily isolated in isolation units) + (0.02 × hospitalized)). It is widely acknowledged that respiratory viruses, such as coronaviruses, have a higher incidence during cooler seasons, especially in temperate regions. Dry and cold conditions during winter are the major drivers for increased respiratory tract infections due to the increased virus stability and transmissibility and weakened host immune system.^19^ Although the respiratory transmission mode of severe acute respiratory syndrome coronavirus 2 (SARS-CoV-​2) is not fully understood, some studies confirmed the seasonality effect.^20,21^ Therefore, we considered a seasonality effect using a sinus function for transmission probability *((((Sin (2 × 3.14 × (Time + 110)/365)) + 1) × ((0.045 – 0.02)/2)) + 0.02)*. The transmission probability was considered 0.02 at the minimum (in June) of the infection wave in the summer and 0.045 at the maximum of the infection wave (in January). Also, the contact rate changed across different time periods, based on the specific interventions introduced in the population at a given time (Table S1 in Supplementary file 1). Individuals who are exposed become infected after *δ*_1_*( Normal( 5.84,0.445))* days.^22^ δ_1_ is the latent period of the disease. We assumed that the incubation period is equal to the latent period and that infected people transmit the infection after the incubation period ends.^23^ Infected individuals are divided into four groups:

- Cases with asymptomatic or mild infection. It is assumed that a proportion of these individuals do not self-isolate and recover at a rate of *α (according to isolation scenario)* after *δ*_8_*( Normal( 10.91,0.50)) *days.^24^
- The remaining cases with asymptomatic or mild infection *( θ( according to isolation scenario))* self-isolate *δ*_6_* (Normal (3,0.5))* days after demonstrating clinical symptoms.
- Cases with severe disease. These individuals are assumed to be referred to the hospital *δ*_2_* (Normal (2,0.5))* days after the onset of symptoms at a rate of *ε( Normal (0.04,0.01)). *
- Cases who die of COVID-19 *δ9 (Normal (11,0.50))* days after the onset of symptoms and before going to the hospital (ω=0.002).

A proportion *( φ( 1-Normal (0.92,0.01)))* of individuals who go to the hospital die after *δ*_3_* (Normal (5,0.5))* days; in addition, a proportion *(ρ(Normal (0.9,0.01)))* of patients who go to hospital proceed to the temporary isolation unit class after *δ*_4_* (Normal (5,0.5))* days. A proportion *(μ( N( 0.995,0.001)))* of individuals who go to temporary isolation units proceed into the recovered class after *δ*_4_*(Normal (7,0.5))* days. The remaining individuals who go to the temporary isolation units *(τ( N( 0.005,0.001)))* die after *δ*_10_*(Normal (7,0.50))* days. Individuals who are in the isolation class proceed to the recovered class after *δ*_7_*(Normal (7.91,0.5))* days. The ordinary differential equations for each of the compartments are shown in Supplementary file 1.

###  Model Parameters, Assumptions, and Calibrations 

 Parameters were obtained and calibrated from the literature, national empirical data, and expert opinion. Our model was also calibrated based on the death toll in Iran to September 21, 2020 (Figure S2 in Supplementary file 1). Due to a lack of sufficient tests and misdiagnosis with other acute respiratory disease, the number of deaths was underreported at the beginning of the epidemic; therefore, death toll underreporting was considered. Parameter descriptions, sources, values, and distributions are presented in the “Mathematical Model” section. Several assumptions were also considered, including:

- Susceptibility of the entire population,
- The incubation period is equal to the latent period and that infected people transmit the infection after the incubation period ends,
- Homogeneity of susceptible and infectious individuals,
- No physical distancing interventions implemented during the early stages of the COVID‐19 epidemic,
- Only 10% self-isolation of infected individuals during the early stages of the COVID‐19 epidemic,
- Negligible migration rates between cities.

Markov Chain Monte Carlo simulation, using a random sample and 10 000 simulations, was performed to calculate the 95% uncertainty intervals (UIs) as plausible bounds around model estimates. These uncertainties were parametrized as probability distributions, based on existing evidence and expert opinion. The 95% UIs were taken as the 2.5^th^ and 97.5^th^ percentiles of the outputs. Data were analyzed using Vensim DSS version 6.4E software. Also, each simulation lasted about one minute.

###  Scenarios for the Impact of Non-pharmaceutical Interventions

 Following the official announcement of the COVID-19 epidemic in Iran, several NPIs were implemented to reduce contact rates among members of the public, as well as to increase self-isolation. In our model, seven scenarios were considered, in which the impacts of changes in contact rates and self-isolation rates were examined. The total number of COVID-19 cases, deaths, and hospitalizations, and number of the existing hospitalized cases at the peak of the epidemic were estimated under each scenario.

*Scenario A:* We assumed that physical distancing interventions and isolation were not implemented. Physical distancing measures included a ban on flights from Wuhan in China, closure of schools and universities, suspension of mass gatherings for religious events, suspension of conferences and social mass gatherings, travel restrictions, cancelation of sporting competitions, closure of some business units in disease epicenters, closure of religious shrines and holy places, and the closure of subways in all cities. We assumed a 50% decrease in contact rates due to behavior changes among the community. Also, we assumed that without any preventive effort the proportion of self-isolation was 10% (Table S1 in Supplementary file 1).

*Scenario B: *Physical distancing measures were assumed to be implemented. However, the proportion of individuals self-isolating was assumed to be 10%.

*Scenario C: *We assumed that physical distancing interventions were not implemented. Self-isolation rates were based on the results of the calibrated model.

*Scenario D: *The impact of physical distancing interventions and self-isolation was evaluated in the context of what would have happened had the health system detected the epidemic in Iran one week earlier, and hence the interventions had started sooner. We also considered three optimistic scenarios if NPIs were increased in Iran.

*Scenario E:* Physical distancing interventions were based on the results of the calibrated model; however, the self-isolation rate was increased to 40%.

*Scenario F:* We considered the self-isolation rate were based on the results of the calibrated model; however, the contact rate was considered to be 8 (we assumed that only work ban interventions were canceled after May 11, 2020 while the other interventions remained).

**Figure 1:**
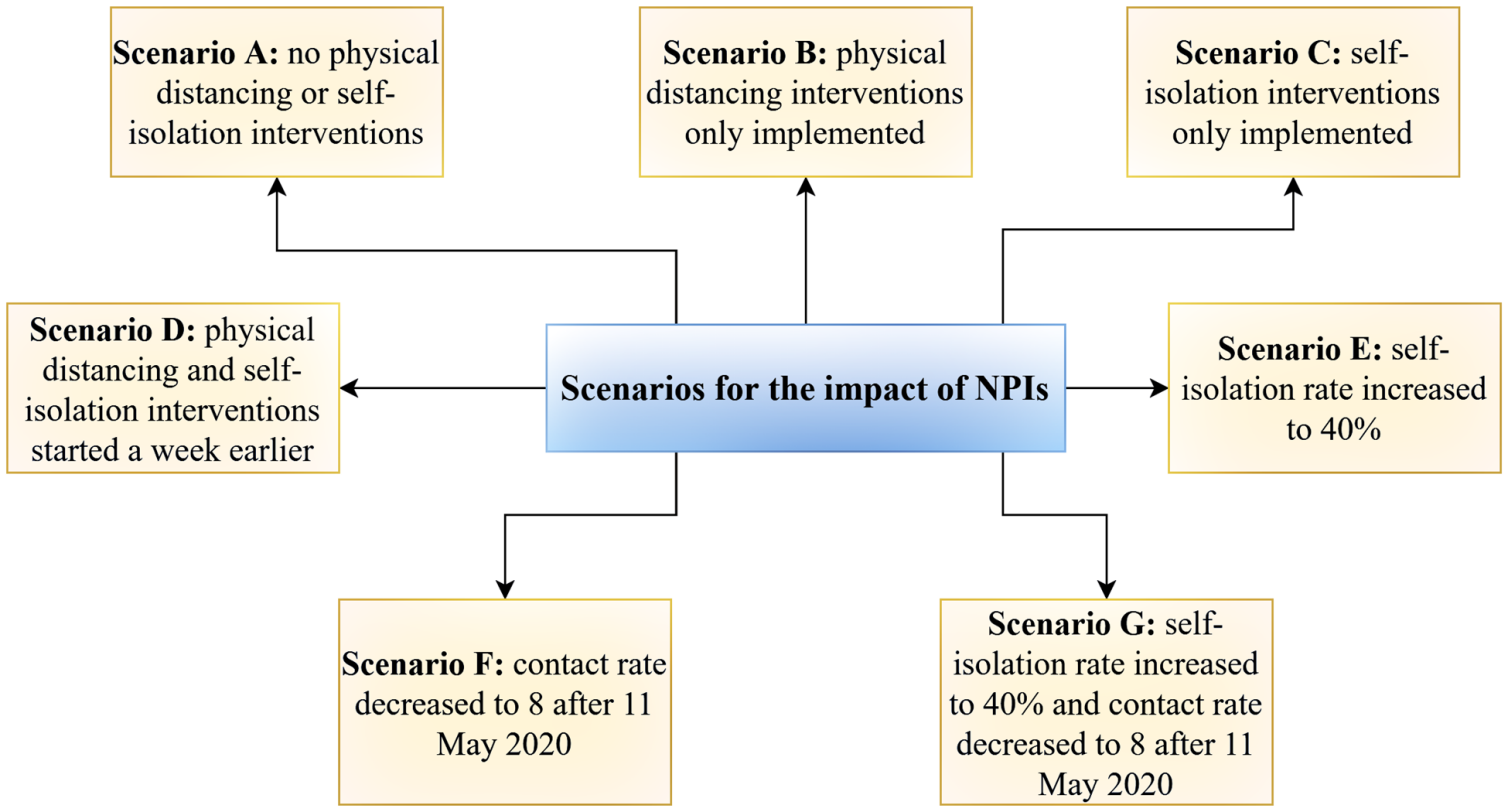
Different Scenarios for the Impact of NPIs in Iran. Abbreviation: NPIs, non-pharmaceutical interventions.

*Scenario G: *We assumed that the self-isolation rate increased to 40% and the contact rate was 8 after May 11, 2020 (Figure 1).

###  Time-Varying Reproduction Number (R_t_ )

 The basic reproduction number (R_0_) is the average number of secondary infections resulting from one infected individual in a susceptible host population. R_0_ is used when there is no immunity from past exposures, vaccinations, or interventions. However, the time-varying reproduction number (R_t_) is used when there are intervention measures. R_t_ can be estimated using mathematical modeling and epidemiologic data. In the current study, we estimated R_t _using both methods. R_t_ was derived using next generation methods with the model that we introduced in previous work.^18^


$$R_{t}=\frac{\beta \left(t\right)*C\left(t\right)}{\left(\frac{\theta }{\delta_{6}}+\frac{\alpha }{\delta_{8}}+\frac{\varepsilon }{\delta_{2}}+\frac{\omega }{\delta_{9}}\right)}$$

To estimate the basic reproductive number from data, we used the daily number of deaths reported by Iran’s Ministry of Health and Medical Education from February 19, to September 21, 2020. A time-dependent method was used to estimate the trend of R_t _in Iran. The mean and standard deviation of serial intervals were considered to be 4.55 and 3.3 days, respectively.^25^ The generation time distribution was considered to be gamma. We analyzed the basic reproductive number using the R_0_ package in R version 4.0.2 software.

## Results

###  Non-pharmaceutical Interventions in Iran

**Figure 2:**
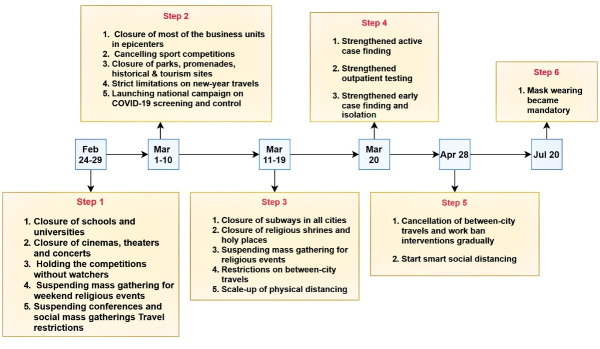
The Schedule of NPIs in Iran. Abbreviations: NPIs, non-pharmaceutical interventions; COVID-19, coronavirus disease 2019.

 A considerable number of interventions were launched and implemented to control the epidemic since the onset of the epidemic. The schedule of NPIs were represented in Figure 2.

###  Calibrated Model

 Under the calibrated model scenario, the total number of deaths up to September 21, 2020 would be 26 000 (95% UI: 3700–91 000). The total number of infected cases predicted in this scenario would be 5 100 000 (95% UI: 680 000–18 000 000). The total number of hospitalized cases and the number of the existing hospitalized cases at the peak of the epidemic would be 420 000 (95% UI: 52 000–1 500 000) and 16 000 (95% UI: 500–70 000), respectively (Table).

**Table:** Comparison of the Total Numbers of Infected and Hospitalized Cases, Deaths, and the Number of the Existing Hospitalized Cases at the Peak, Under Different Scenarios, up to September 21, 2020

	**Calibrated ** **Model**	**Reported Data**	**Scenarios**						
			**A**	**B**	**C**	**D**	**E**	**F**	**G**
Number of deaths (95% UI)	26 000 (3700-91 000)	24 478	266 000 (119 000–476 000)	155 000 (24 000–360 000)	127 000 (26 000–282 000)	18 000 (2600–70 000)	13 000 (2600–41000)	17 000 (3600–55 000)	10 000 (3000–26 000)
Number of infected cases (95% UI)	5 100 000 (680 000–18 000 000)	-	51 800 000 (19 100 000–77 600 000)	29 200 000 (4 800 000–58 700 000)	25 400 000 (5 900 000–51 700 000)	3 300 000 (430 000–13 200 000)	2 300 000 (400 000–7 200 000)	2 800 000 (599 000–9 500 000)	1 700 000 (511 000–4 800 000)
Number of hospitalized cases (95% UI)	420 000 (52 000–1 500 000)	425 481	4 800 000 (1 600 000–9 500 000)	3 000 000 (476 000–6 800 000)	2 190 000 (424 000–5 100 000)	312 000 (39 000–1 190 000)	223 000 (43 000–780 000)	284 000 (55 000–924 000)	163 000 (44 000–463 000)
Number of the existing hospitalized cases at the peak (95% UI)	16 000 (500–70 000)	-	360 000 (112 000–650 000)	153 000 (17 000–350 000)	114 000 (16 000–260 000)	13 000 (2000–67 000)	10 000 (3500–20 000)	13 000 (5000–28 000)	11 000 (4000–24 000)

Abbreviation: UI, uncertainty interval. **Scenario A: **no physical distancing or self-isolation interventions; **Scenario B: **physical distancing interventions only implemented; **Scenario C: **self-isolation interventions only implemented;** Scenario D:** physical distancing and self-isolation interventions started a week earlier;** Scenario E: **self-isolation rate increased to 40%; **Scenario F: **contact rate decreased to 8 after May 11, 2020; **Scenario G:** self-isolation rate increased to 40% and contact rate decreased to 8 after May 11, 2020.

###  Scenario A

 Under scenario A, the death toll by September 21, 2020 would be 266 000 (95% UI: 119 000–476 000). It is estimated that the total number of infected cases under scenario A would be 51 800 000 (95% UI: 19 100 000–77 600 000). Also, the total number of hospitalized cases in this scenario would be 4 800 000 (95% UI: 1 600 000–9 500 000). At the peak of epidemic, there would be 360 000 (95% UI: 112 000–650 000) existing cases in hospital (Table).

###  Scenario B

 The death toll in scenario B was expected to be 155 000 (95% UI: 24 000–360 000). The total number of infected cases would be 29 200 000 (95% UI: 4 800 000–58 700 000). It is estimated that the total number of hospitalized cases and the number of the existing hospitalized cases at the peak would be 3 000 000 (95% UI: 476 000–6 800 000) and 153 000 (95% UI: 17 000–350 000), respectively (Table).

###  Scenario C

 Under this scenario, the total number of death cases up to September 21, 2020 would be 127 000 (95% UI: 26 000–282 000). It is predicted that the total number of infected cases under scenario C would be 25 400 000 (95% UI: 5 900 000–51 700 000). The total number of hospitalized cases and the number of the existing hospitalized cases at the peak would be 2 190 000 (95% UI: 424 000–5 100 000) and 114 000 (95% UI: 16 000–260 000), respectively (Table).

###  Scenario D

 Under scenario D, the death toll up to September 21, 2020 would be 18 000 (95% UI: 2600–70 000). It is estimated that the total number of infected cases under scenario D would be 3 300 000 (95% UI: 430 000–13 200 000). The total number of hospitalized cases in this scenario would be 312 000 (95% UI: 39 000–1 190 000). Also, the number of the existing hospitalized cases at the peak would be 13 000 (95% UI: 2000–67 000) (Table).

###  Scenario E

 Under this scenario, the death toll up to September 21, 2020 would be 13 000 (95% UI: 2600–41 000). It is predicted that the total number of infected cases under scenario E would be 2 300 000 (95% UI: 400 000–7 200 000). The total number of hospitalized cases and the number of the existing hospitalized cases at the peak would be 223 000 (95% UI: 43 000-780 000) and 10 000 (95% UI: 3500–20 000), respectively (Table).

###  Scenario F

 The death toll in scenario F was expected to be 17 000 (95% UI: 3600–55 000). It is estimated that the total number of infected cases would be 2 800 000 (95% UI: 599 000-9 500 000). The total number of hospitalized cases would be 284 000 (95% UI: 55 000–924 000). Also, the number of the existing hospitalized cases at the peak would be 13 000 (95% UI: 5000–28 000) (Table).

###  Scenario G

 Under scenario G, the death toll up to September 21, 2020 was expected to be 10 000 (95% UI: 3000–26 000). The total number of infected cases under scenario G would be 1 700 000 (95% UI: 511 000–4 800 000). It is predicted that the total number of hospitalized cases and the number of the existing hospitalized cases at the peak would be 163 000 (95% UI: 44 000–463 000) and 11 000 (95% UI: 4000–24 000), respectively (Table).

**Figure 3:**
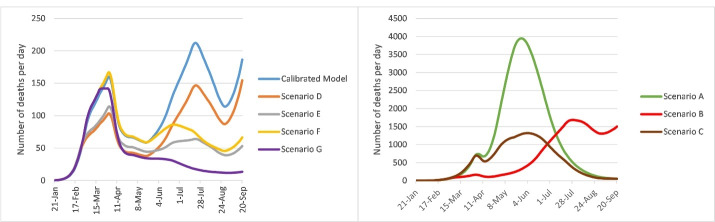
The Estimated Death toll in Iran Under Different Scenarios, From January 21 to September 21, 2020.

**Figure 4:**
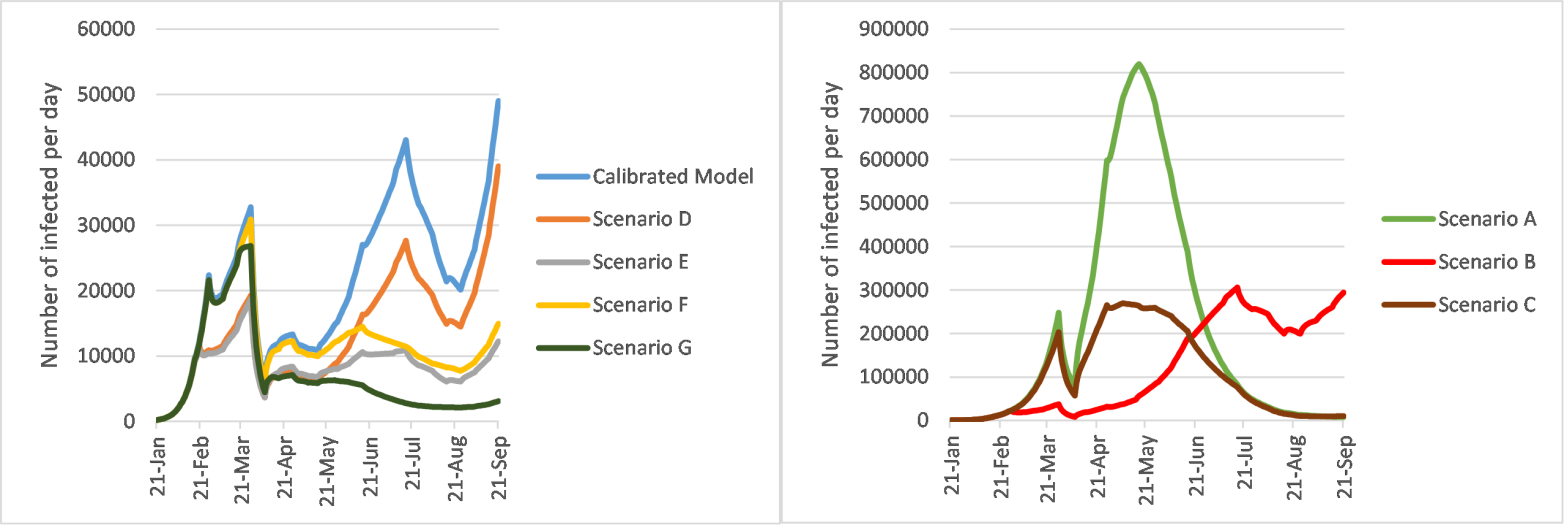
The Estimated Number of Infected Cases Per Day in Iran Under Different Scenarios From January 21, to September 21, 2020.

**Figure 5:**
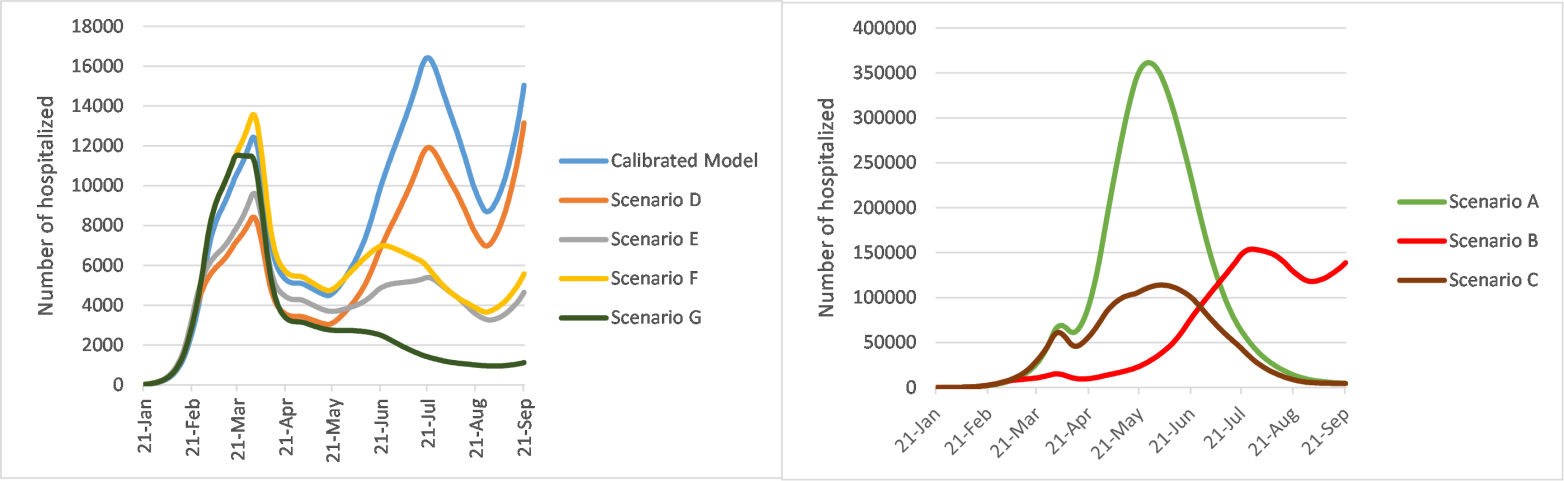
The Estimated Number of Existing Hospitalized Cases in Iran Under Different Scenarios From January 21, 2020 to September 21, 2020.

 The estimated death tolls, numbers of infected cases per day, and numbers of existing hospitalized cases under the different scenarios from January 21 to September 21, 2020 are shown in Figures 3, 4, and 5.

###  Impact of Interventions on Reproductive Numbers

**Figure 6:**
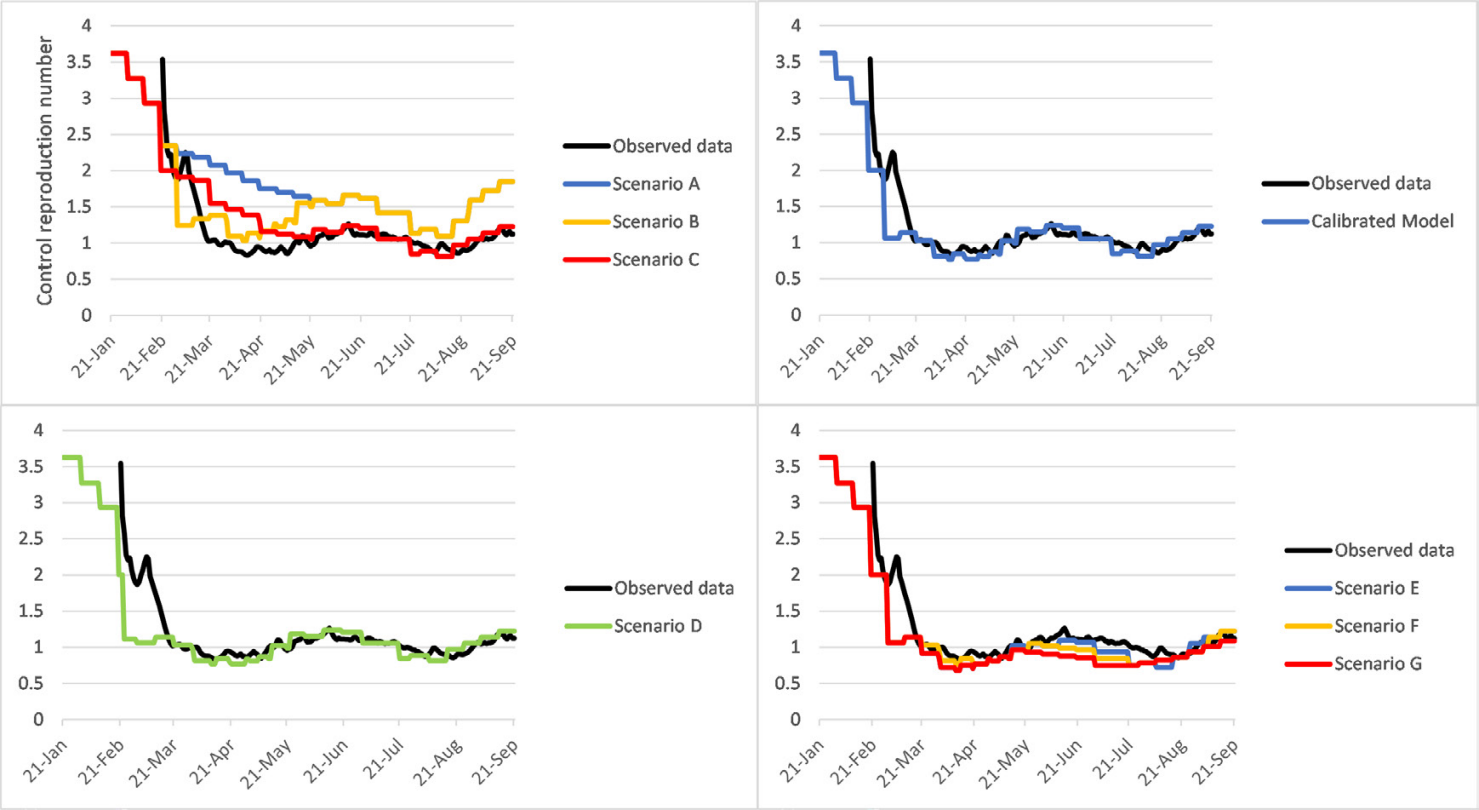
Comparison of the Daily Reproduction Number Using the Time-Dependent Method and Different Scenarios From January 21, 2020 to September 21, 2020.

 At the beginning of the epidemic, the R_t_ in Iran was estimated to be more than 3. After the implementation of various interventions, R_t_ decreased to less than one from April 5 to May 10, 2020. Unfortunately, after May 11, the R_t_ increased again, to more than one. The R_t_ was more than 1.26 from June 6, to June 15, 2020 (Figure 6). The R_t_ decreased to near 1 in July then increased to more than one from mid-August to September 21, 2020.

## Discussion

 Iran is in a challenging position, having a population with a relatively high average age and therefore a relatively large proportion of the population at risk of severe COVID-19 disease. Limits to the health system capacity mean that there is a continuous risk of breaching this capacity. Given the economic constraints and the abundance of multigenerational households, shielding of the elderly is not a viable component of any scenario for Iran. Economic constraints also limit the feasible levels of self-isolation of infected individuals to a range of between 10% and 40%. This means that neither containment nor shielding approaches are realistic strategy options in Iran. Maintaining a balance between deaths caused by COVID-19, as explored with this modeling approach, and deaths caused through economic hardship resulting from COVID-19 interventions (which are beyond the scope of this article), while avoiding breaches in health system capacity, is therefore a continuing challenge for the Iranian government, until a vaccine or other health technologies become available.

 As the COVID-19 pandemic progressed, countries increasingly implemented a broad range of responses. Our results demonstrate that the multiple interventions performed in Iran have had a profound effect in mitigating the epidemic. Our results also showed that the interventions resulted in an average self-isolation rate of 30% of the population.

 Our results showed that without strict social distancing and self-isolation measures there would have been a considerably higher number of infections and deaths, as much as ten-times higher than currently. It is widely understood that suppression of the epidemic will require the use of more intensive and socially disruptive measures. Suppression may not be a feasible target in all countries, as the choice of interventions and their intensity ultimately depends on the infrastructure required and the relative feasibility of these measures in different social contexts.

 It should be noted that self-isolation, social distancing, and travel restrictions will have a profound effect, reducing the workforce across many economic sectors and causing many jobs to be lost. However, the effect of opening low-risk jobs that have minimum interference with the above control measures seems to have little effect on the number of infections and deaths. Economic anxiety and economic crisis are currently considered to be two major side effects of the COVID-19 pandemic. This economic crisis and anxiety will be more disruptive in resource-limited countries.^26^ Iran, as a developing country, needs resilient and strong leadership in healthcare, business, government, and wider society to manage the financial challenges presented by the COVID-19 pandemic. Immediate relief measures may need to be adjusted for workers who may otherwise fall through the cracks. Medium- and longer-term strategies to re-balance the economy will also be needed following this crisis.^27^

 Our study found that the number of infections and deaths would not decrease significantly if the self-isolation rates were increased or contact rates were decreased from those achieved by the measures actually implemented in Iran. This suggests the interventions already implemented in the country were effective.

 Our results also demonstrated that if the control measures in Iran had been started just 7 days earlier, the total number of deaths and infections would have decreased by 30%. This would also have resulted in a reduction in the number of hospitalizations at the peak by 20%. These findings have important implications for any second and third waves of the epidemic, either in Iran or other countries, and highlight the necessity for countries to develop early-warning systems as soon as possible. The use of triggers based on hospital admissions might be a more efficient early-warning system in settings where extended and ongoing community-based testing is not in place. In countries where testing coverage and consistency is not homogenous across the country, local early-warning triggers based on community-testing results might be an efficient method in localities where there are comprehensive and ongoing community-testing activities. Local time-series analyses that provide correlations and time-lags between infections and hospitalizations would also help in warning hospitals to be prepared.

 To avoid any rebound in transmission, these policies will need to be maintained until large stocks of vaccine are available to immunize the population. When this might be remains unclear. Until then, early-warning systems can be used as a guide for policy-makers, helping them to adjust their control measures in the population.

 Our model indicated that if minimum (ie, only school closures, border closures, work bans, or event bans) or no interventions were implemented, the number of hospitalizations with COVID-19 would exceed hospital bed capacity. As of April 2020, public hospitals in Iran, which are mainly responsible for the COVID-19 response, had around 150 000 beds, 9000 of which were in intensive care units. As of April 2020, there were 0.41 physicians and 1.14 nurses per public hospital bed in Iran. This implies that a shortage of healthcare workers would be challenging in this scenario and even in scenarios with more interventions. Given that a proportion of healthcare workers would be infected, isolated, and even die due to complications of COVID-19, the shortage of healthcare workers would become even worse, especially under scenarios where no intervention or minimum intervention is considered.

 Our projections suggest that the measures introduced by the government of Iran resulted in large (around 60%) reductions in the total number of contacts. The observed reduction appears unlikely to have been due to chance, given the large difference in the average R_t_. This is consistent with recent studies conducted in Wuhan, China, as well as in the UK, that respectively estimated an 85% and 74% reduction in the average number of daily contacts under physical distancing interventions.^28,29^ This is also in line with the results of Khosravi et al and Aghaali et al, who reported a gradual decrease in R_t_ over time in Shohroud and Qom (central Iran).^25,30^ The gradual decrease in R_t_ observed in our study is promising and further highlights the possible effectiveness of NPIs implemented in Iran. Some of these measures included public education to promote social distancing and self-isolation at home.

 There are three main limitations to this study. First, any of the properties of the virus that causes COVID-19 remain unknown, thus some of the data we used may have uncertainties. However, to address these uncertainties, we used the Markov Chain Monte Carlo method and reported the UIs. Applying these uncertainties in several variables lead to wide UIs. Second, we also made several assumptions; these were mentioned in the methods section. However, these assumptions may not be exactly true in the real world. Third, we assumed that the incubation period is equal to the latent period. Based on this assumption, infected people could transmit the infection after the incubation period ends. However, some studies explained the virus could transmit before the incubation period ends and pre-symptomatic people could transmit the virus.^31,32^

## Conclusion

 The effectiveness of NPIs may vary by country or community, depending on the extent of community engagement and the quality of the interventions. Our models suggest that the NPIs implemented in Iran between January 21, and September 21, 2020 had a satisfactory effect on reducing the number of COVID-19 infections and deaths in the country. Therefore, it is recommended that these interventions continue to be implemented by the government. Given the economic restrictions on Iran imposed by US sanctions, however, it is highly recommended that innovative modifications and solutions are considered that could minimize the economic impact of these NPIs on individuals and the state. Examples may include use of mobile money and microfinance to drive small business development, strengthening insurance and other adaptation measures to enhance resilience, strengthening digitalization of businesses, and direct involvement of the government in supporting innovative solutions. Our modeling also highlighted the profound effect the timely implementation of NPIs can have on further reductions in the number of infections and deaths. This reinforces the importance of forecasting and the early detection of future waves of the epidemic through mathematical modeling studies, as well as the development of early-warning systems. Proper preparedness and timely responses to any future waves of disease could be achieved through such systems.

## Acknowledgements

 We would like to acknowledge the contributions of supervisors who provided inputs to the study design and methods, and assisted in implementation of the surveys. We also would like to acknowledge the editorial services were provided by Adam Bodley.

## Ethical issues

 Ethical approval for the research was received from the Kerman University of Medical Sciences, Kerman, Iran (reference 98001239).

## Competing interests

 AAH is the Deputy Minister of Education and the Head of National Committee on COVID-19 Epidemiology. The rest of the authors declare no conflict of interest, real or perceived.

## Authors’ contributions

 In this work MN, SE, and HS took the lead, were responsible for the data analysis and made substantial contributions to conception, design, and writing. YJ and MAG made substantial contributions to conception, design, and writing. HS, LW, and AAH revised the study critically, contributed substantially to conception, design, the interpretation of data, and drafting of the article.

## Supplementary files

Supplementary file 1. Ordinary Differential Equations and Conceptual Model.

## References

[1] Zhu N, Zhang D, Wang W (2020). A novel coronavirus from patients with pneumonia in China, 2019. N Engl J Med.

[2] World Health Organization. Coronavirus disease (COVID-19) pandemic.Available from: https://www.who.int/emergencies/diseases/novel-coronavirus-2019. Accessed November 27, 2020.

[3] Times NY. Iran reports its first 2 cases of the new coronavirus. https://www.timesofisrael.com/iran-reports-its-first-2-cases-of-the-new-coronavirus/. Accessed May 9, 2020.

[4] Arab-Mazar Z, Sah R, Rabaan AA, Dhama K, Rodriguez-Morales AJ (2020). Mapping the incidence of the COVID-19 hotspot in Iran - Implications for Travellers. Travel Med Infect Dis.

[5] CDC NCoC-EaI. Analysis of epidemic trend by provinces of Iran. http://corona.behdasht.gov.ir/files/site1/files/IRAN_COVID19_Factsheet_N.58_-19September_En.pdf. Accessed September 19, 2020.

[6] COVID-19 Coronavirus. https://www.worldometers.info/coronavirus/coronavirus-cases/. Accessed September 19, 2020.

[7] National Committee on COVID-19 Epidemiology and Iranian CDC - Ministry of Health and Medical Education II. Major interventions to control COVID-19 from 19 Feb. http://corona.behdasht.gov.ir/files/site1/files/1584533009223_Major_interventions_to_control_COVID-19_from.pdf. Accessed March 15, 2020.

[8] Abdi M (2020). Coronavirus disease 2019 (COVID-19) outbreak in Iran: actions and problems. Infect Control Hosp Epidemiol.

[9] Davies NG, Kucharski AJ, Eggo RM, Gimma A, Edmunds WJ (2020). Effects of non-pharmaceutical interventions on COVID-19 cases, deaths, and demand for hospital services in the UK: a modelling study. Lancet Public Health.

[10] Yang J, Zhang Q, Cao Z (2021). The impact of non-pharmaceutical interventions on the prevention and control of COVID-19 in New York City. Chaos.

[11] Lai S, Ruktanonchai NW, Zhou L (2020). Effect of non-pharmaceutical interventions to contain COVID-19 in China. Nature.

[12] Bo Y, Guo C, Lin C (2021). Effectiveness of non-pharmaceutical interventions on COVID-19 transmission in 190 countries from 23 January to 13 April 2020. Int J Infect Dis.

[13] Ngonghala CN, Iboi E, Eikenberry S (2020). Mathematical assessment of the impact of non-pharmaceutical interventions on curtailing the 2019 novel coronavirus. Math Biosci.

[14] Hens N, Vranck P, Molenberghs G (2020). The COVID-19 epidemic, its mortality, and the role of non-pharmaceutical interventions. Eur Heart J Acute Cardiovasc Care.

[15] Childs ML, Kain MP, Kirk D, et al. The impact of long-term non-pharmaceutical interventions on COVID-19 epidemic dynamics and control. medRxiv. 2020. 10.1101/2020.05.03.20089078. PMC8385372 34428971

[16] Awaidy SA, Mahomed O (2020). Impact of non-pharmaceutical interventions on the COVID-19 epidemic: a modelling study. SAGE Open Med.

[17] Bajiya VP, Bugalia S, Tripathi JP (2020). Mathematical modeling of COVID-19: impact of non-pharmaceutical interventions in India. Chaos.

[18] Sharifi H, Jahani Y, Mirzazadeh A, et al. Estimating COVID-19-related infections, deaths, and hospitalizations in Iran under different physical distancing and isolation scenarios. Int J Health Policy Manag. 2020. 10.34172/ijhpm.2020.134. PMC9278464 32772007

[19] Moriyama M, Hugentobler WJ, Iwasaki A (2020). Seasonality of respiratory viral infections. Annu Rev Virol.

[20] Petersen E, Koopmans M, Go U (2020). Comparing SARS-CoV-2 with SARS-CoV and influenza pandemics. Lancet Infect Dis.

[21] Shi P, Dong Y, Yan H (2020). Impact of temperature on the dynamics of the COVID-19 outbreak in China. Sci Total Environ.

[22] Khalili M, Karamouzian M, Nasiri N, Javadi S, Mirzazadeh A, Sharifi H (2020). Epidemiological characteristics of COVID-19: a systematic review and meta-analysis. Epidemiol Infect.

[23] Kucharski AJ, Russell TW, Diamond C (2020). Early dynamics of transmission and control of COVID-19: a mathematical modelling study. Lancet Infect Dis.

[24] You C, Deng Y, Hu W (2020). Estimation of the time-varying reproduction number of COVID-19 outbreak in China. Int J Hyg Environ Health.

[25] Aghaali M, Kolifarhood G, Nikbakht R, Mozafar Saadati H, Hashemi Nazari SS (2020). Estimation of the serial interval and basic reproduction number of COVID-19 in Qom, Iran, and three other countries: a data-driven analysis in the early phase of the outbreak. Transbound Emerg Dis.

[26] COVID-19: Disrupting lives, economies and societies. Available from: https://www.un.org/development/desa/dpad/publication/world-economic-situation-and-prospects-april-2020-briefing-no-136/. Accessed April 1, 2020.

[27] Nicola M, Alsafi Z, Sohrabi C (2020). The socio-economic implications of the coronavirus pandemic (COVID-19): a review. Int J Surg.

[28] Zhang J, Litvinova M, Liang Y, et al. Age profile of susceptibility, mixing, and social distancing shape the dynamics of the novel coronavirus disease 2019 outbreak in China. medRxiv. 2020. 10.1101/2020.03.19.20039107.

[29] Jarvis CI, Van Zandvoort K, Gimma A (2020). Quantifying the impact of physical distance measures on the transmission of COVID-19 in the UK. BMC Med.

[30] Khosravi A, Chaman R, Rohani-Rasaf M, Zare F, Mehravaran S, Emamian MH (2020). The basic reproduction number and prediction of the epidemic size of the novel coronavirus (COVID-19) in Shahroud, Iran. Epidemiol Infect.

[31] He X, Lau EHY, Wu P (2020). Temporal dynamics in viral shedding and transmissibility of COVID-19. Nat Med.

[32] Tindale LC, Coombe M, Stockdale JE, et al. Transmission interval estimates suggest pre-symptomatic spread of COVID-19. medRxiv. 2020. 10.1101/2020.03.03.20029983.

