# Supplementary material for: Impact of Non-pharmaceutical Interventions on the Control of COVID-19 in Iran: A Mathematical Modeling Study
Source: Int J Health Policy Manag. 2021 Jun 9;11(8):1472–81. doi: 10.34172/ijhpm.2021.48 (PMC9808365; doi:10.34172/ijhpm.2021.48)
Supplement: Supplementary file 1 — Ordinary Differential Equations and Conceptual Model. [file ijhpm-11-1472-s001.pdf]

Article title: Impact of Non-pharmaceutical Interventions on the Control of COVID-19 in Iran: A Mathematical Modeling Study

Journal name: International Journal of Health Policy and Management (IJHPM)

Authors' information: Mehran Nakhaeizadeh[1,2]¶, Sana Eybpoosh[3]¶, Yunes Jahani[1,2], Milad Ahmadi Gohari1,[2], Ali Akbar Haghdoost[1,4], Lisa White[5], Hamid Sharifi[4,2]*

[1]Modeling in Health Research Center, Institute for Futures Studies in Health, Kerman University of Medical Sciences, Kerman, Iran.

[2]Department of Biostatistics and Epidemiology, School of Public Health, Kerman University of Medical Sciences, Kerman, Iran.

[3]Department of Epidemiology and Biostatistics, Research Centre for Emerging and Reemerging Infectious Diseases, Pasteur Institute of Iran, Tehran, Iran.

[4]HIV/STI Surveillance Research Center, and WHO Collaborating Center for HIV Surveillance, Institute for Futures Studies in Health, Kerman University of Medical Sciences, Kerman, Iran.

[5]Big Data Institute, Li Ka Shing Centre for Health Information and Discovery, Nuffield Department of Medicine, University of Oxford, Oxford, UK.

¶Both authors contributed equally to this paper.

(*Corresponding author: hsharifi@kmu.ac.ir)

Supplementary file 1. Ordinary Differential Equations and Conceptual Model

The ordinary differential equations of the compartments are as follows:

$$\frac{dS}{dt} = -\beta(t)C(t)\frac{II(t)}{N}S$$

$$\frac{dE}{dt} = \beta(t)C(t)\frac{II(t)}{N}S - \frac{1}{\delta_1}E$$

$$\frac{dI}{dt} = \frac{1}{\delta_1}E - \left(\frac{\theta}{\delta_6} + \frac{\alpha}{\delta_8} + \frac{\varepsilon}{\delta_2} + \frac{\omega}{\delta_9}\right)I$$

$$\frac{dR}{dt} = \frac{\mu}{\delta_5}T + \frac{\alpha}{\delta_8}I + \frac{1}{\delta_7}I_s$$

$$\frac{dI_s}{dt} = \frac{\theta}{\delta_6}I - \frac{1}{\delta_7}I_s$$

$$\frac{dH}{dt} = \frac{\varepsilon}{\delta_2}I - (\frac{\varphi}{\delta_3} + \frac{\rho}{\delta_4})H$$

$$\frac{dT}{dt} = \frac{\rho}{\delta_4}H - (\frac{\mu}{\delta_5} + \frac{\tau}{\delta_{10}})T$$

$$\frac{dD}{dt} = \frac{\varphi}{\delta_3}H + \frac{\omega}{\delta_9}I + \frac{\tau}{\delta_{10}}T$$

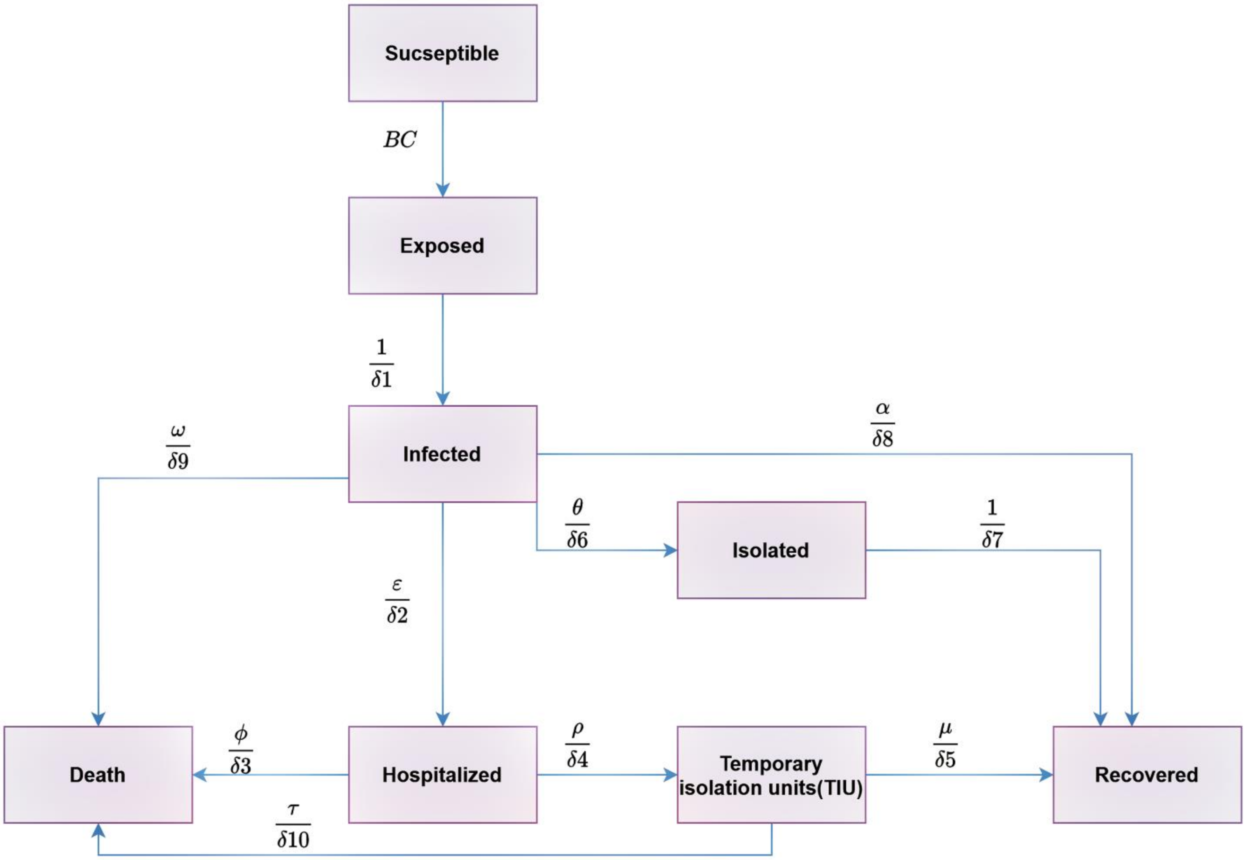

Figure S1. The SEIR conceptual model we used to assess impact of non-pharmaceutical interventions on the control of COVID-19 in Iran

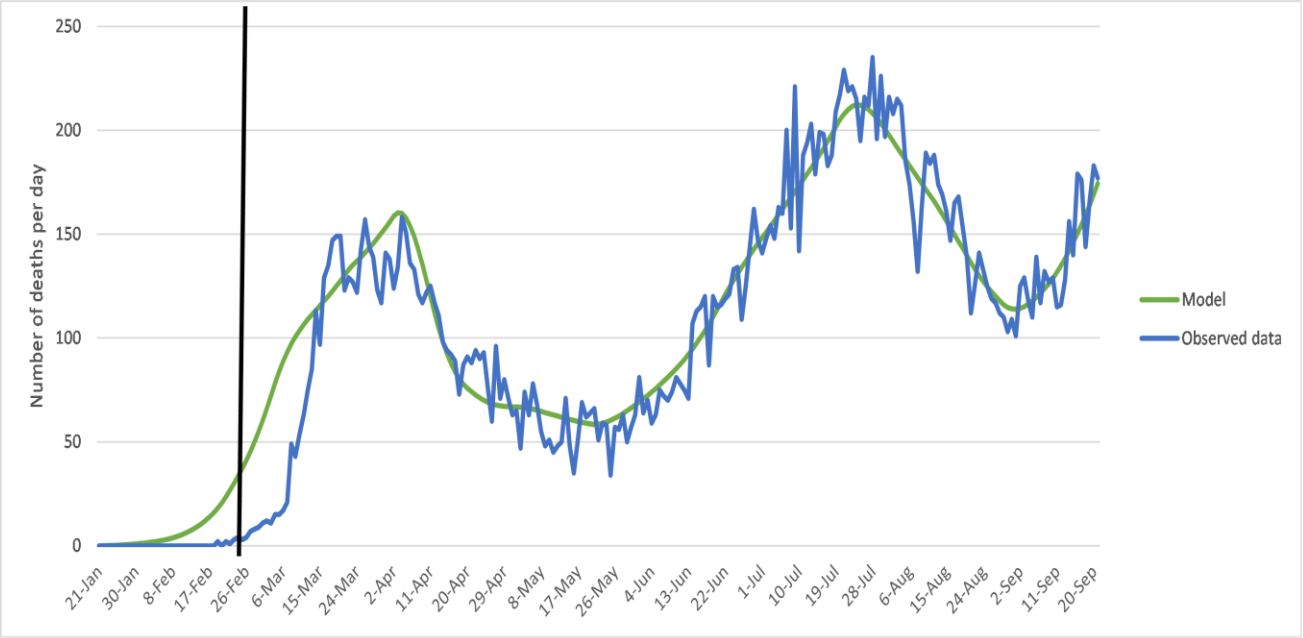

Figure S2. The calibrated model output based on the death toll in Iran. (The vertical black line is the date when the NPIs were started in Iran).

Table S1. Contact rates (per participant per day) and self-isolation rates for different scenarios from 21 January to 21 September 2020

**Date**	**Scenario**															
	Calibrated model		**A**		**B**		**C**		**D**		**E**		**F**		**G**	
	**C**	**I**	**C**	**I**	**C**	**I**	**C**	**I**	**C**	**I**	**C**	**I**	**C**	**I**	**C**	**I**
From Jan 21, 2020 to Jan 30, 2020	13	10	13	10	13	10	13	10	13	10	13	10	13	10	13	10
From Jan 31, 2002 to Feb 9, 2020	12	10	12	10	12	10	12	10	12	10	12	10	12	10	12	10
From Feb 10, 2020 to Feb 19, 2020	11	10	11	10	11	10	11	10	11	10	11	10	11	10	11	10
From Feb 20, 2020 to Feb 22, 2020	9	20	9	10	9	10	9	20	9	20	9	20	9	20	9	20
From Feb 23, 2020 to Feb 29, 2020	9	20	9	10	9	10	9	20	5	20	9	20	9	20	9	20
From Mar 1, 2020 to Mar 10, 2020	5	20	9	10	5	10	9	20	5	20	5	20	5	20	5	20
From Mar 11, 2020 to Mar 12, 2020	5.5	20	9	10	5.5	10	9	20	5.5	20	5.5	20	5.5	20	5.5	20
From Mar 13, 2020 to Mar 20, 2020	5.5	20	9	10	5.5	10	9	20	5.5	20	5.5	20	5.5	20	5.5	20
From Mar 21, 2020 to Mar 31, 2020	6	30	9	10	6	10	9	30	6	30	6	40	6	30	6	40
From Apr 1, 2020 to Apr 12, 2020	5	30	9	10	5	10	9	30	5	30	5	40	5	30	5	40
From Apr 13, 2020 to Apr 20, 2020	5.5	30	9	10	5.5	10	9	30	5.5	30	5.5	40	5.5	30	5.5	40
From Apr 21, 2020 to Apr 28, 2020	6	40	9	10	6	10	9	40	6	40	6	40	6	40	6	40
From Apr 28, 2020 to May 5, 2020	6.5	40	9	10	6.5	10	9	40	6.5	40	6.5	40	6.5	40	6.5	40
From May 6, 2020 to May 12, 2020	7	40	9	10	7	10	9	40	7	40	7	40	7	40	7	40
From May 13, 2020 to May 22, 2020	8.5	40	9	10	8.5	10	9	40	8.5	40	8.5	40	8	40	8	40
From May 23, 2020 to Jun 9, 2020	9	30	9	10	9	10	9	30	9	30	9	40	8	30	8	40
From Jun 10, 2020 to Jun 30, 2020	10	30	10	10	10	10	10	30	10	30	10	40	8	30	8	40
From Jul 1, 2020 to Jul 20, 2020	10	30	10	10	10	10	10	30	10	30	10	40	8	30	8	40
From Jul 21, 2020 to Aug 5, 2020	8	40	8	10	8	10	8	40	8	40	8	40	8	40	8	40
From Aug 6, 2020 to Aug 15, 2020	7	40	7	10	7	10	7	40	7	40	7	40	8	40	8	40
From Aug 16, 2020 to Aug 25, 2020	8	40	8	10	8	10	8	40	8	40	8	40	8	40	8	40
From Aug 26, 2020 to Sep 5, 2020	9	40	9	10	9	10	9	40	9	40	9	40	8	40	8	40
From Sep 27, 2020 to Sep 21, 2020	9	30	9	10	9	10	9	30	9	30	9	40	8	30	8	40

C = contact rate (per participant per day), I = percentage self-isolation rate
